# Supplementary material for: Presynaptic accumulation of α-synuclein causes synaptopathy and progressive neurodegeneration in Drosophila
Source: Brain Commun. 2021 Mar 22;3(2):fcab049. doi: 10.1093/braincomms/fcab049 (PMC8111063; doi:10.1093/braincomms/fcab049)
Supplement: fcab049_Supplementary_Data [file fcab049_supplementary_data.pdf]

Presynaptic accumulation of α-synuclein causes synaptopathy and progressive neurodegeneration in *Drosophila*

Jessika C. Bridi[1#]*, Erika Bereczki[4], Saffron K. Smith[5], Gonçalo M. Poças[3,6], Benjamin Kottler[1], Pedro M. Domingos[3], Christopher J. Elliott[5], Dag Aarsland[2,4], Frank Hirth[1]*

* Correspondence to:
Frank Hirth: Frank.Hirth@kcl.ac.uk
Jessika Bridi: jessikabridi@usp.br/jessikacbridi@gmail.com

This file includes:

- Figures 1 and 2;
- Tables 1- 4;
- Full-size western blotting membrane images;
- Detailed Materials and Methods.

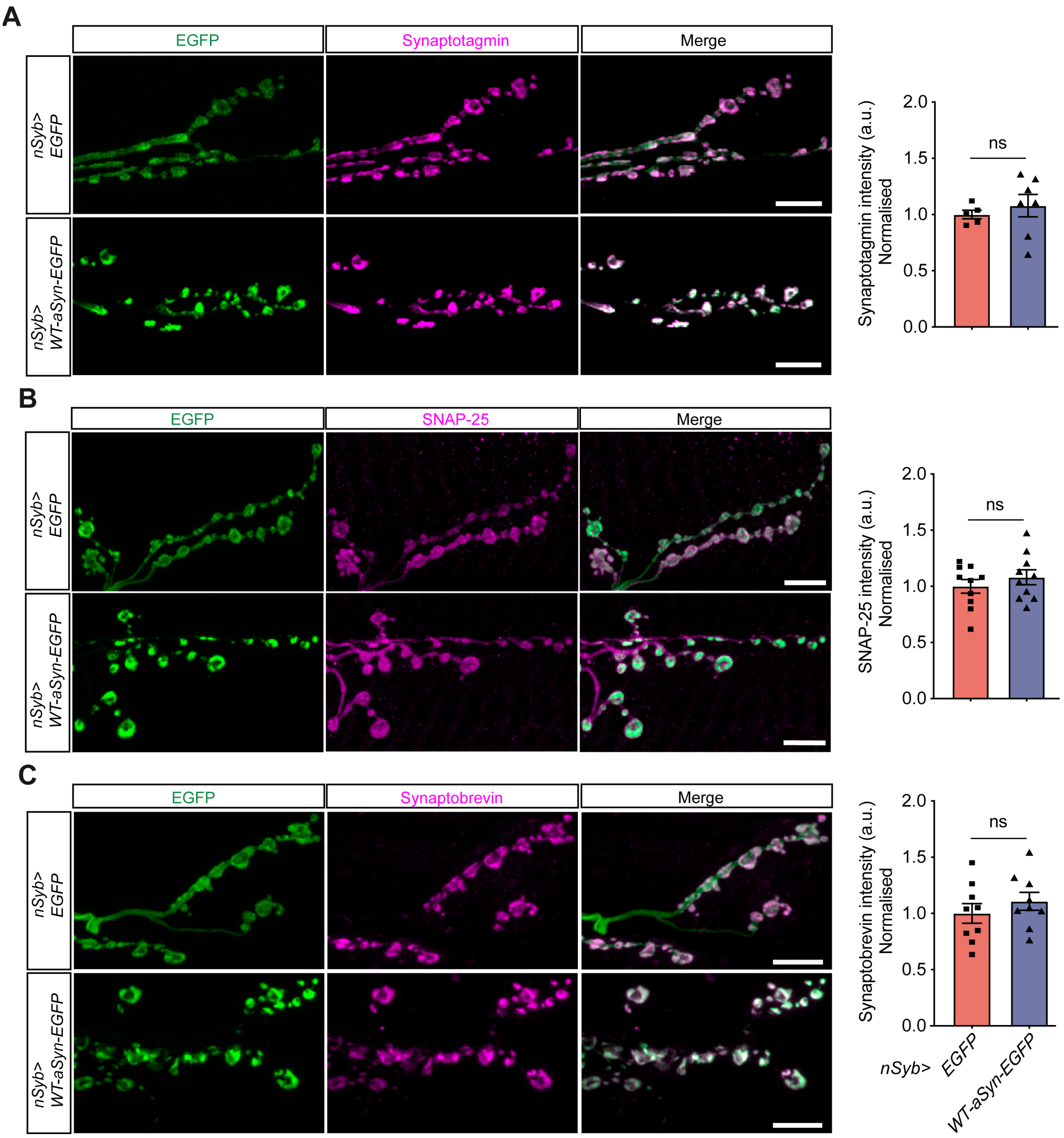

Fig. 1. Presynaptic accumulation of α-Syn causes specific presynaptic deficits. Representative confocal images of NMJ immunolabeled with anti-Synaptotagmin (A), anti-SNAP-25 (B), anti-nSynaptobrevin (C) and anti-GFP at the NMJ shows they are unaffected. Quantification of fluorescence intensity showed that WT-α-Syn expression under control of the pan-neuronal driver *nSyb-Gal4* caused no alterations in these proteins compared to control group expressing GFP only; ns – not significant $p>0.05$; n=5-10 NMJ from 5/7 flies (A), 10 flies (B), and 8 flies (C)/genotype. Mean ± SEM are shown; statistical analyses were performed using unpaired t test. Scale bars: 10 µm.

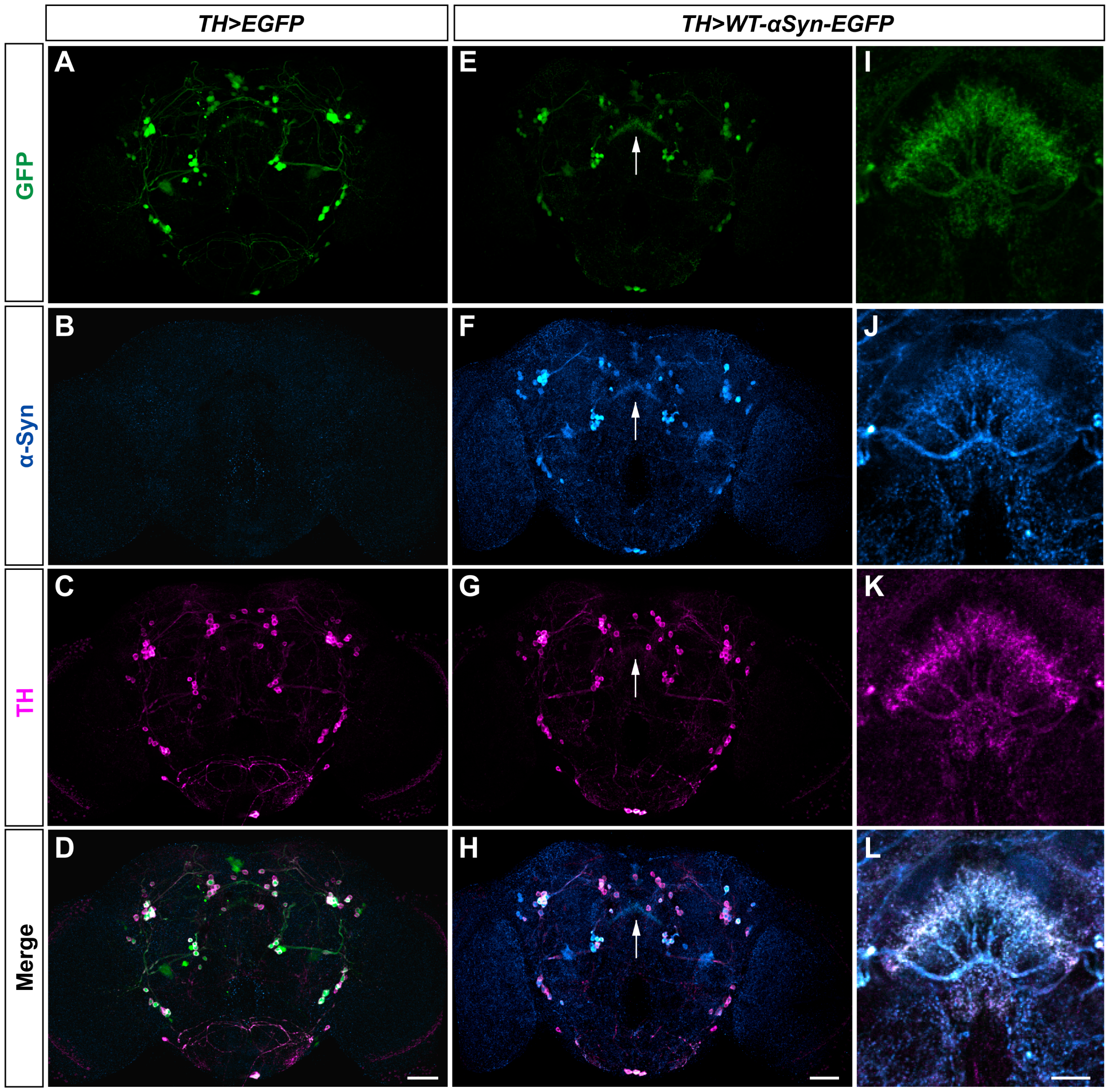

Fig. 2. Expression of α-Syn in the dopaminergic system of the adult *Drosophila* brain. (A-D) EGFP control or (E-L) WT-α-Syn-EGFP expression in 3-day-old flies under control of *TH-Gal4* driver; adult brains were immunolabelled with anti-GFP (green), anti- α-Syn (blue) and anti-TH (magenta). Note, absence of α-Syn in the control *TH>EGFP* (B) compared to the experimental group expressing WT-α-syn-EGFP labelled with anti-α-Syn (F, J). (C, G, J) Dopaminergic neurons, their projections and synaptic terminals in the control and experimental group labelled with anti-TH. (D, H, L) Merge of anti-GFP, anti-α-Syn and anti-TH labelling; arrowhead indicates synaptic terminals within the fan-shaped body of the central complex; scale bar = 50 μm. (I-L) Higher magnification of immunolabelled fan-shaped body reveals that α-syn also accumulates in synaptic terminals; scale bar = 20 μm.

Table 1: Quantification of phenotypes and statistical tests – Figures 1-8.

Figure		Genotype	n number	Mean	Std. Deviation	Std. Error of the mean	Statistical tests			
**Figure 1**	B	*nSyb>EGFP*	5	1.137	0.329	0.147	Unpaired two-tailed t-test	t(9)=7.720, p<0.0001		
		nSyb>WT-α-Syn-EGFP	6	2.339	6.379	21.604				
**Figure 2**	B	*nSyb>EGFP*	6	1.000	0.146	0.059	Unpaired two-tailed t-test	t(10)=2.639, p=0.0248		
		nSyb>WT-α-Syn-EGFP	6	0.808	0.156	0.063				
	D	*nSyb>EGFP*	12	1.000	0.2813	0.081		t(23)=10.45, p<0.0001		
		nSyb>WT-α-Syn-EGFP	13	0.163	0.064	0.017				
**Figure 3**	B - 3-day-old	*nSyb>EGFP*	7	1.011	0.053	0.020	Unpaired two-tailed t-test	t(14)=0.5700, p=0.5777		
	B - 20-day-old	*nSyb>EGFP*	9	1.000	0.024	0.008				
	C - 3-day-old	*nSyb>WT-α-Syn-EGFP*	8	1.255	0.066	0.023		t(14)=2.250, p=0.0411		
	C - 20-day-old	*nSyb>WT-α-Syn-EGFP*	8	1.394	0.162	0.057				
	E - 3-day-old	*1-nSyb>EGFP*	3	1.000	0.003	0.002	Two-way ANOVA (mixed effect)	Genotype [F (1,14) = 28.98, p<0.0001]		
	E - 3-day-old	*2-nSyb>WT-α-Syn-EGFP*	3	0.681	0.172	0.100		Age [F (1,14) = 1.470, p=0.2455]		
	E - 20-day-old	*3- nSyb>EGFP*	6	1.000	0.098	0.040		Šídák's multiple comparisons test (genotype)	1 vs. 2	Adj. p=0.0024
	E - 20-day-old	*4- nSyb>WT-α-Syn-EGFP*	6	0.798	0.068	0.028			3 vs. 4	Adj. p=0.0056
	G - 3-day-old	*1-nSyb>EGFP*	3	1.000	0.113	0.065	Two-way ANOVA (mixed effect)	Genotype [F (1,10) = 8.762, p<0.0143]		
	G - 3-day-old	*2-nSyb>WT-α-Syn-EGFP*	3	0.892	0.017	0.010		Age [F (1,4) = 0.2256, p=0.6595]		
	G - 20-day-old	*3- nSyb>EGFP*	6	1.000	0.068	0.028		Šídák's multiple comparisons test (genotype)	1 vs. 2	Adj. p=0.2079
	G - 20-day-old	*4- nSyb>WT-α-Syn-EGFP*	6	0.867	0.075	0.031			3 vs. 4	Adj. p=0.0151
**Figure 4**	C	*1- nSyb/+*	8	1028.0	122.2	43.20	One-way ANOVA [F(2,22)= 5.205, p=0.0141]	Tukey's multiple comparison post-hoc test	1 vs. 2	Adj. p=0.9028
		2- nSyb>EGFP	8	1009	65.21	23.05			1 vs. 3	Adj. p=0.0189
		3- nSyb>WT-α-Syn-EGFP	9	899.3	71.26	23.75			2 vs. 3	Adj. p=0.0492
	D	*1- nSyb/+*	8	1.533	0.224	0.079	One-way ANOVA		1 vs. 2	Adj. p=0.9998

		2- nSyb>EGFP	8	1.531	0.260	0.092	[F(2,22)= 5.188, p=0.0143]		1 vs. 3	Adj. p=0.0289
		3- nSyb>WT-α-Syn-EGFP	9	1.236	0.172	0.057			2 vs. 3	Adj. p=0.0300
	E	*1- nSyb/+*	8	130.600	22.18	7.840	One-way ANOVA [F(2,22)= 1.795, p=0.1897]		1 vs. 2	Adj. p=0.9835
		2- nSyb>EGFP	8	129.000	18.62	6.585			1 vs. 3	Adj. p=0.2250
		3- nSyb>WT-α-Syn-EGFP	9	115.700	12.27	4.122			2 vs. 3	Adj. p=0.2976
**Figure 5**	E	*1- TH/+*	9	8.309	2.559	0.853	One-way ANOVA [F (2,19) = 4.859, p=0.0198]	Tukey's multiple comparison post-hoc test	1 vs. 2	Adj. p=0.9892
		2- TH>EGFP	7	8.115	2.141	0.809			1 vs. 3	Adj. p=0.0313
		3-TH>WT-α-Syn-EGFP	6	12.310	3.553	1.450			2 vs. 3	Adj. p=0.0325
	F	*1-TH/+*	11	112.900	19.130	5.768	One-way ANOVA [F (2,20) = 0.2507, p=0.7807]		1 vs. 2	Adj. p=0.7909
		2- TH>EGFP	6	106.600	9.114	3.721			1 vs. 3	Adj. p=0.9998
		3- TH>WT-α-Syn-EGFP	6	113.100	24.970	10.190			2 vs. 3	Adj. p=0.8261
**Figure 6**	B - Top	*1- TH/+*	100	28.880	11.030	1.103	Kruskal-Wallis test	Dunn's multiple comparison post-hoc test	1 vs. 2	Adj. p=0.0596
		2- WT-α-Syn-EGFP/+	89	25.870	9.572	1.015			1 vs. 3	Adj. p<0.0001
		3- TH>WT-α-Syn-EGFP	100	21.240	9.151	0.915			2 vs. 3	Adj. p=0.0071
	B - Bottom	*1- TH/+*	30	26.930	9.364	1.710			1 vs. 2	Adj. p>0,9999
		2- WT-α-Syn-EGFP/+	30	28.920	10.550	1.926			1 vs. 3	Adj. p=0.0002
		3- TH>WT-α-Syn-EGFP	30	16.290	7.953	1.452			2 vs. 3	Adj. p<0.0001
	C - Top	*1- TH/+*	100	4.657	0.668	0.067			1 vs. 2	Adj. p<0,9999
		2- WT-α-Syn-EGFP/+	89	4.623	0.725	0.077			1 vs. 3	Adj. p<0.0001
		3- TH>WT-α-Syn-EGFP	100	4.178	0.509	0.051			2 vs. 3	Adj. p<0.0001
	C - Bottom	*1- TH/+*	30	4.731	0.682	0.124			1 vs. 2	Adj. p<0.9999
		2- WT-α-Syn-EGFP/+	30	4.831	0.679	0.124			1 vs. 3	Adj. p<0.0001
		3- TH>WT-α-Syn-EGFP	30	3.805	0.387	0.071			2 vs. 3	Adj. p<0.0001
	E- Top	*1- TH/+*	100	0.509	0.214	0.021			1 vs. 2	Adj. p=0.0165
		2- WT-α-Syn-EGFP/+	89	0.438	0.162	0.017			1 vs. 3	Adj. p<0.0001
		3- TH>WT-α-Syn-EGFP	100	0.382	0.164	0.016			2 vs. 3	Adj. p=0.1795
	E - Bottom	*1- TH/+*	30	0.436	0.158	0.029			1 vs. 2	Adj. p<0.9999
		2- WT-α-Syn-EGFP/+	30	0.469	0.162	0.030			1 vs. 3	Adj. p=0.0097

		3- TH>WT-α-Syn-EGFP	30	0.304	0.156	0.029			2 vs. 3	Adj. p=0.0011
	F - Top	*1- TH/+*	100	0.734	0.190	0.019			1 vs. 2	Adj. p=0.432
		2- WT-α-Syn-EGFP/+	89	0.698	0.200	0.021			1 vs. 3	Adj. p<0.0001
		3- TH>WT-α-Syn-EGFP	100	0.608	0.140	0.014			2 vs. 3	Adj. p=0.0053
	F- Bottom	*1- TH/+*	30	0.801	0.186	0.034			1 vs. 2	Adj. p<0.9999
		2- WT-α-Syn-EGFP/+	30	0.802	0.162	0.029			1 vs. 3	Adj. p<0.0001
		3- TH>WT-α-Syn-EGFP	30	0.555	0.095	0.017			2 vs. 3	Adj. p<0.0001
	G - Top	*1- TH/+*	100	2.158	1.015	0.105			1 vs. 2	Adj. p=0.0017
		2- WT-α-Syn-EGFP/+	89	2.572	1.129	0.121			1 vs. 3	Adj. p<0.0001
		3- TH>WT-α-Syn-EGFP	100	3.504	2.787	0.280			2 vs. 3	Adj. p=0.1102
	G - Bottom	*1- TH/+*	30	2.871	1.820	0.332			1 vs. 2	Adj. p<0.9999
		2- WT-α-Syn-EGFP/+	30	2.587	1.438	0.263			1 vs. 3	Adj. p=0.0108
		3- TH>WT-α-Syn-EGFP	30	5.243	5.675	1.036			2 vs. 3	Adj. p=0.0014
	K - 3-day-old	*1- nSyb/+*	12	91.790	6.162	1.779	One-way ANOVA [F (2, 35) =7.480, p=0.0020]		1 vs. 2	Adj. p=0.004
		2- nSyb>EGFP	13	97.380	2.873	0.797			1 vs. 3	Adj. p=0.0066
		3- nSyb>WT-α-Syn-EGFP	13	97.080	2.100	0.583			2 vs. 3	Adj. p=0.9794
	K - 10-day-old	*1- nSyb/+*	10	94.400	4.222	1.335	One-way ANOVA [F (2,27) =1.093, p=0.3496]		1 vs. 2	Adj. p=0.3239
		2- nSyb>EGFP	10	97.200	4.638	1.467			1 vs. 3	Adj. p=0.848
		3- nSyb>WT-α-Syn-EGFP	10	95.450	3.947	1.248			2 vs. 3	Adj. p=0.6359
	K - 20-day-old	*1- nSyb/+*	10	78.800	8.651	2.736	One-way ANOVA [F (2, 26) = 33.71, p<0.0001]	Tukey's multiple comparison post-hoc test	1 vs. 2	Adj. p=0.5097
		2- nSyb>EGFP	9	84.670	9.434	3.145			1 vs. 3	Adj. p<0.0001
		3- nSyb>WT-α-Syn-EGFP	10	45.400	14.850	4.696			2 vs. 3	Adj. p<0.0001
	K - 30-day-old	*1- nSyb/+*	10	80.220	9.732	3.078	One-way ANOVA [F (2, 27) = 49.95, p<0.0001]		1 vs. 2	Adj. p=0.7789
		2- nSyb>EGFP	10	83.600	7.589	2.400			1 vs. 3	Adj. p<0.0001
		3- nSyb>WT-α-Syn-EGFP	10	38.800	14.880	4.706			2 vs. 3	Adj. p<0.0001
	K - 40-day-old	*1- nSyb/+*	9	44.440	17.290	5.762	One-way ANOVA [F (2, 24) = 16.71, p<0.0001]		1 vs. 2	Adj. p=0.6703
		2- nSyb>EGFP	9	50.440	17.830	5.942			1 vs. 3	Adj. p=0.0004
		3- nSyb>WT-α-Syn-EGFP	9	12.890	6.412	2.137			2 vs. 3	Adj. p<0.0001

	O	*1- TH/+*	14	57.830	8.117	2.169	One-way ANOVA [F (2, 38) = 14.35, p<0.0001]		1 vs. 2	Adj. p=0.0275
		2- TH>EGFP	13	44.510	13.870	3.847			1 vs. 3	Adj. p<0.0001
		3- TH>WT-α-Syn-EGFP	14	31.840	15.430	4.124			2 vs. 3	Adj. p=0.0377
**Figure 7**	C - PPL1 (3-day-old)	*1- TH/+*	28	11.250	0.928	0.175	One-way ANOVA [F (2, 79) = 0.6142, p=0.5437]	Tukey's multiple comparison post-hoc test	1 vs. 2	Adj. p=0.9285
		2- TH>EGFP	26	11.120	1.033	0.203			1 vs. 3	Adj. p=0.5223
		3- TH>WT-α-Syn-EGFP	28	10.860	1.860	0.352			2 vs. 3	Adj. p=0.7619
	C - PPL1 (20-day-old)	*1- TH/+*	25	11.600	0.764	0.153	One-way ANOVA [F (2, 90) = 7.474, p=0.0010]		1 vs. 2	Adj. p=0.6676
		2- TH>EGFP	31	11.320	1.077	0.193			1 vs. 3	Adj. p=0.0016
		3- TH>WT-α-Syn-EGFP	37	10.490	1.502	0.247			2 vs. 3	Adj. p=0.0146
	C - PPL1 (40-day-old)	*1- TH/+*	32	11.380	0.942	0.167	One-way ANOVA [F (2, 94) = 4,522, p=0.0133]		1 vs. 2	Adj. p=0.959
		2- TH>EGFP	32	11.280	1.054	0.186			1 vs. 3	Adj. p=0.0207
		3- TH>WT-α-Syn-EGFP	33	10.450	1.872	0.326			2 vs. 3	Adj. p=0.0425
	D - PPM3 (3-day-old)	*1- TH/+*	28	6.357	1.393	0.263	One-way ANOVA [F (2, 78) = 0.7740, p=0.4647]		1 vs. 2	Adj. p=0.8349
		2- TH>EGFP	25	6.120	1.453	0.291			1 vs. 3	Adj. p=0.4313
		3- TH>WT-α-Syn-EGFP	28	5.857	1.649	0.312			2 vs. 3	Adj. p=0.8013
	D - PPM3 (20-day-old)	*1- TH/+*	25	6.600	1.258	0.252	One-way ANOVA [F (2, 93) = 3,429, p=0.0366]		1 vs. 2	Adj. p=0.2869
		2- TH>EGFP	34	7.059	0.886	0.152			1 vs. 3	Adj. p=0.6808
		3- TH>WT-α-Syn-EGFP	37	6.351	1.274	0.209			2 vs. 3	Adj. p=0.0292
	D - PPM3 (40-day-old)	*1- TH/+*	32	6.875	0.907	0.160	One-way ANOVA [F (2, 95) = 3.497, p=0.0342]		1 vs. 2	Adj. p=0.7808
		2- TH>EGFP	32	6.625	1.996	0.353			1 vs. 3	Adj. p=0.0333
		3- TH>WT-α-Syn-EGFP	34	5.941	1.369	0.235			2 vs. 3	Adj. p=0.155
	E - PPL2 (3-day-old)	*1- TH/+*	28	8.429	0.997	0.189	One-way ANOVA [F (2, 75) = 0.5210, p=0.5961]		1 vs. 2	Adj. p=0.9682
		2- TH>EGFP	23	8.522	1.039	0.217			1 vs. 3	Adj. p=0.728
		3- TH>WT-α-Syn-EGFP	27	8.148	1.854	0.357			2 vs. 3	Adj. p=0.602
	E - PPL2 (20-day-old)	*1- TH/+*	25	8.400	1.155	0.231	One-way ANOVA [F (2, 91)= 0.0121, p=0.9879]		1 vs. 2	Adj. p=0.9989
		2- TH>EGFP	34	8.382	1.706	0.293			1 vs. 3	Adj. p=0.988
		3- TH>WT-α-Syn-EGFP	35	8.343	1.434	0.242			2 vs. 3	Adj. p=0.9932
	E - PPL2 (40-day-	*1- TH/+*	32	8.813	0.821	0.145	One-way ANOVA [F (2,		1 vs. 2	Adj. p=0.9479

	old)	*2- TH>EGFP*	31	8.742	0.729	0.131	92) = 0.1727, p=0.8417]		1 vs. 3	Adj. p=0.9583
		3- TH>WT-α-Syn-EGFP	32	8.875	1.100	0.194			2 vs. 3	Adj. p=0.8271
	F - PPM1/2 (3-day-old)	*1- TH/+*	28	8.214	0.630	0.119	One-way ANOVA [F (2,79) = 2.602, p=0.0805]		1 vs. 2	Adj. p=0.9953
		2- TH>EGFP	26	8.231	0.652	0.128			1 vs. 3	Adj. p=0.1114
		3- TH>WT-α-Syn-EGFP	28	8.571	0.690	0.130			2 vs. 3	Adj. p=0.145
	F - PPM1/2 (20-day-old)	*1- TH/+*	26	8.038	1.216	0.239	One-way ANOVA [F (2, 96) = 1.001, p=0.3715]		1 vs. 2	Adj. p=0.3977
		2- TH>EGFP	35	8.429	0.884	0.149			1 vs. 3	Adj. p=0.4506
		3- TH>WT-α-Syn-EGFP	38	8.395	1.326	0.215			2 vs. 3	Adj. p=0.9915
	F - PPM1/2 (40-day-old)	*1- TH/+*	32	8.875	0.421	0.074	One-way ANOVA [F (2, 95) = 3.251, p=0.0431]		1 vs. 2	Adj. p=0.0752
		2- TH>EGFP	32	8.438	0.840	0.149			1 vs. 3	Adj. p=0.0728
		3- TH>WT-α-Syn-EGFP	34	8.441	0.991	0.170			2 vs. 3	Adj. p=0.9998

Table 2: Human active zone core proteins in PDD and DLB patient's vs Control

Uniprot Accession nr	Protein name		PDD vs C		DLB vs C	
			FC	p value	FC	p value
Q86UR5	RIMS1	RIM 1-4	0.85	0.216	1.08	0.790
Q9UQ26	RIMS2		0.89	0.131	1.02	0.9434
Q9UJD0	RIMS3		1.12	0.070	1.10	0.119
Q9H426	RIMS4		0.89	0.660	0.81	0.509
Q9Y6V0	PCLO	PICCOLO	0.88	0.204	1.07	0.710
Q8IUD2	RB6l2/ERC1	ELKS/CAST	0.99	0.784	0.94	0.249
Q13136	LIPA1	LIPRINα 1-4	0.99	0.923	0.94	0.572
O75334	LIPA2		0.96	0.827	0.92	0.635
O75145	LIPA3		0.85	0.182	0.84	0.125
O75335	LIPA4		0.83	0.131	0.73	**0.0185**

Abbreviations: RIM: regulating synaptic membrane exocytosis; PCLO: piccolo presynaptic cytomatrix protein; RB6l2/ ERC1: ELKS/RAB6-interacting/CAST family member 1; LIPA: PTPRF interacting protein alpha; FC: fold change.

Table 3: Demographics and clinical characteristics of the subjects included in the study.

Diagnose	Age	Gender M/F	Braak stage	MMSE score
**C**	78.2±7.5	6/4	1 (0-1)	NA
**DLB**	81.5±7.6	4/6	3 (1-5)	13.4±10.2
**PDD**	79.6±3.8	5/5	2 (1-5)	15.9±9.2

Age at death are mean values, MMSE is the median score before death and Braak stage is median, both with range in brackets. There was no significant difference between diagnostic groups with regards to age or gender. Pathological and cognitive scores of DLB patients were significantly worse than of C (Braak stage p=0.009) or PDD (MMSE p=0.031) patients. Abbreviations: C-control; DLB-dementia with Lewy bodies; PDD-Parkinson`s disease with dementia; MMSE-mini mental state examination; NA- not available.

Table 4: Quantification of phenotypes and statistical tests – Figure 8.

**LIPRIN-α3 – Fig 8A**							
	BA9 (prefrontal cortex)			BA24 (cingulate cortex)		BA40 (parietal cortex)	
	Control	PDD	DLB	Control	DLB	Control	DLB
Number of patients	10	10	10	10	10	10	10
Mean	1.102	0.8868	1.001	1.081	1.092	1.273	1.118
Std. Deviation	0.3690	0.2963	0.3631	0.4141	0.3472	0.4958	0.2631
Std. Error of the mean	0.1167	0.0937	0.1148	0.1309	0.1098	0.1568	0.0832
Statistical tests	One-way ANOVA [F (2.27) =0.9780, p=0.39]			Unpaired two-tailed t-test		Unpaired two-tailed t-test	
	Dunnett´s multiple comparison post-hoc test						
	Control vs. PDD		Adj. p= 0.2909	t(18)=0.068, p= 0.9462		t(18)=0.878, p= 0.3914	
	Control vs. DLB		Adj. p= 0.7397				
**LIPRIN-α4 – Fig 8B**							
	BA9 (prefrontal cortex)			BA24 (cingulate cortex)		BA40 (parietal cortex)	
	Control	PDD	DLB	Control	DLB	Control	DLB
Number of patients	10	8	10	10	10	10	10
Mean	1.620	1.480	1.276	1.530	1.207	1.140	1.115
Std. Deviation	0.2977	0.4031	0.2116	0.4442	0.4384	0.3130	0.3687
Std. Error of the mean	0.0941	0.1425	0.0669	0.1405	0.1386	0.0989	0.1166
Statistical tests	One-way ANOVA [F (2.25) =3.189, p=0.05]			Unpaired two-tailed t-test		Unpaired two-tailed t-test	
	Dunnett´s multiple comparison post-hoc test						
	Control vs. PDD		Adj. p=0.5355	t(18)=1.636, p=0.1192		t(18)=0.161, p=0.8737	
	Control vs. DLB		Adj. p= 0.0349				

Full-Size western blotting membrane images

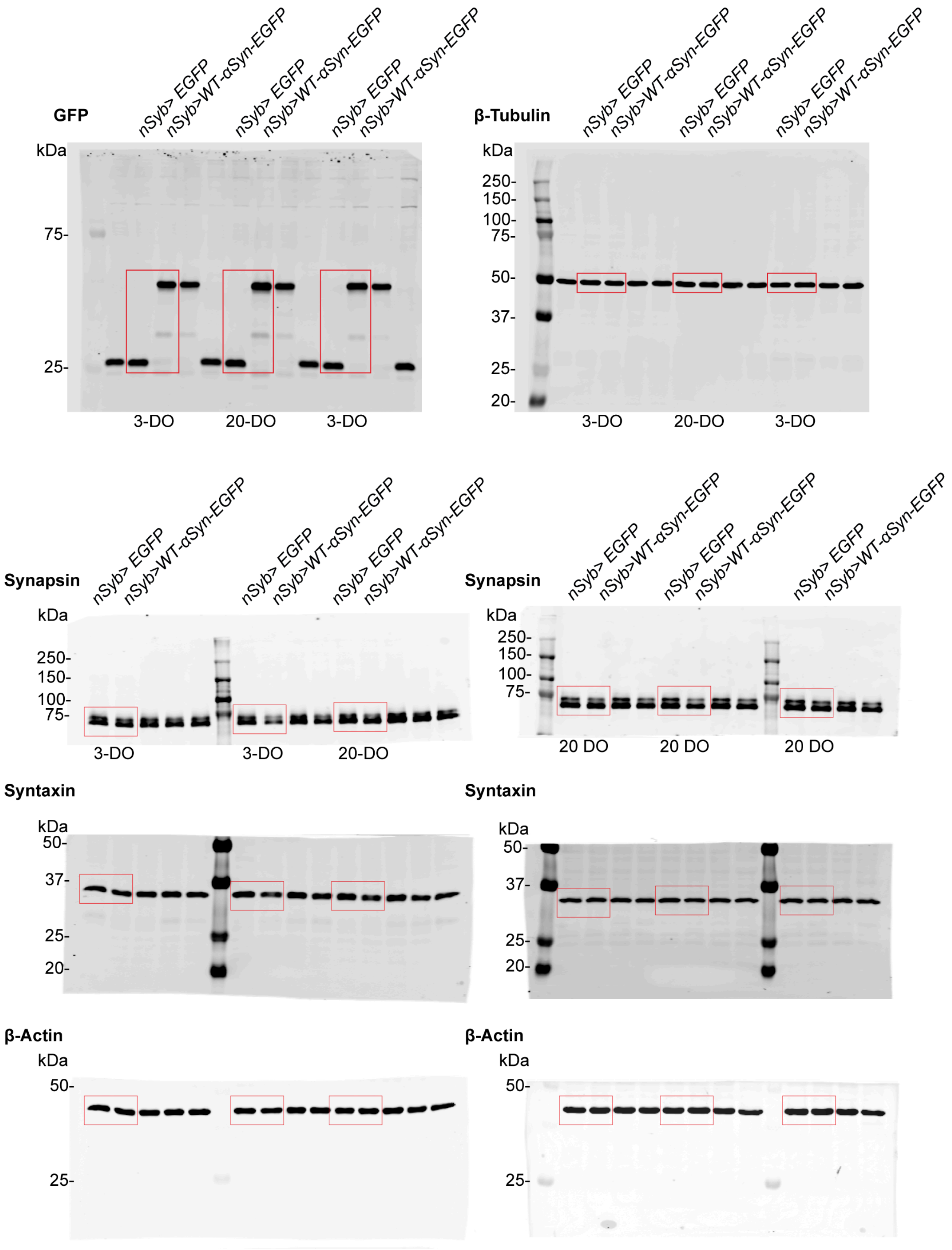

Figure 3 A-C - Full size western blotting membranes.

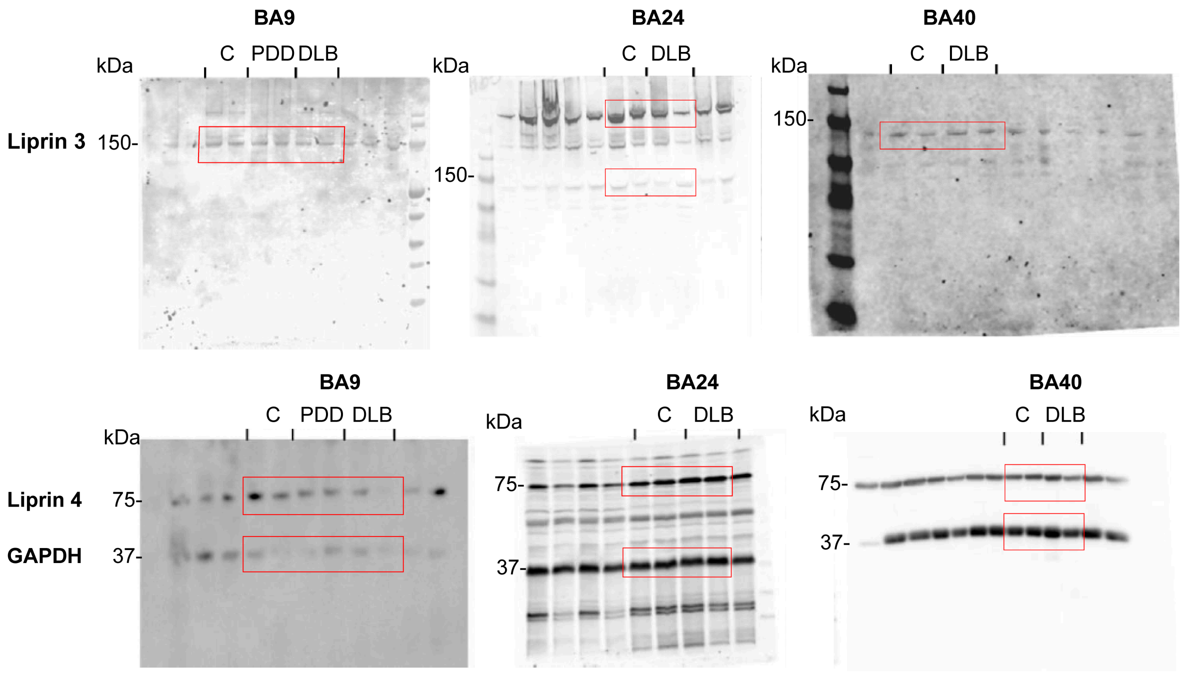

Figure 8A-C - Full size western blotting membranes.

Detailed Materials and Methods

Fly stocks and husbandry

All fly stocks were maintained in standard cornmeal media at 25°C in a 12 h light/dark cycle, unless for ageing experiments where flies were kept in 15% yeast/sugar media [1–3]. Strain used were *Oregon R, W^{1118}, nSyb-gal4 (a kind gift from Dr Sean Sweeney), TH-gal4* [4]*, UAS-EGFP, UAS-WT-α-syn-EGFP* [5]

Immunofluorescence

Drosophila larval NMJ dissections were carried out according to established protocol [6] and fixed either with 3.5% formaldehyde for 25 min or Bouin's fixative (Sigma) for 5 min. Samples were blocked with 10% normal goat serum (Thermo Fisher Scientific) for 20 min and incubated with primary antibody. Primary antibodies used were anti-HRP (1:200 - Immunochemicals 123-605-021), anti-CSP (1:200 - DSHB), anti-Synapsin (1:50 - DSHB), anti-nSynaptobrevin (1:150 - Ohyama *et al.*, 2007; a kind gift from Dr Hugo Bellen, Baylor College of Medicine), anti-Synaptotagmin (1:1000 – West *et al.*, 2015; a kind gift from Dr Sean Sweeney, University of York), anti-SNAP-25 (1:100 - Rao *et al.*, 2001; a kind gift from Dr David Deitcher, Cornell University), anti-GFP (1:500 - Thermo Fischer A6455), anti-BRP (1:50 - DSHB). Adult CNS preparations were carried out as described previously [3]. The primary antibodies used were anti-TH (1:50 - ImmunoStar), anti-GFP (1:500 - Thermo Fischer Scientific A6455). Secondary antibodies were Alexa fluor 488 and 568 (1:150; Invitrogen).

Imaging and analysis

Z-stacks of NMJ synapses innervating muscle 6/7 of segment 3 were captured with a Nikon A1R confocal microscope with Nikon Plan Apochromat 60x NA 1.40 oil-immersion

objective or Leica TCS SP5 equipped with HCX Plan Apochromat 63.0x NA 1.40 oil-immersion. The adult *Drosophila* brain images were acquired using Nikon Plan Apochromat 20x NA 0.75 objective for DA neuron cluster analysis. The instant super resolution structured illumination microscopy (iSIM) was performed using Nikon Eclipse Ti-E Inverted microscope with 100x 1.49 NA to image both for adult CNS and NMJ preparations.

For the fluorescence quantification, to build up the ratio between GFP signal in the synaptic boutons and axons, the intensity of ten synaptic boutons (labelled with anti-CSP) and ten axonal regions (positive for anti-HRP and negative for anti-CSP) were quantified per NMJ. Thus, each n number represents the average value obtained from the division of fluoresce intensity of synaptic boutons/axon in each NMJ. For fluorescence quantifications of Synapsin and CSP, z-stacks were obtained using identical settings for all genotypes with same z-axis spacing between them within the same experiment and optimised for detection without saturation of the signal[10]. Ten synaptic boutons were analysed per NMJ using the free hand tool from ImageJ (http://imagej.nih.gov/ij/), with each point in the graphs representing the average of ten synaptic boutons/NMJ.

BRP puncta number were manually counted in z-stacks using ImageJ and the Cell Counter plugin (developed by Dr Kurt De Vos, available at https://imagej.nih.gov/ij/plugins/cell-counter.html) to record the total number of puncta per NMJ. Synapse surface area was calculated by creating a mask around the HRP channel, that labels the neuronal membrane, using ImageJ thresholding and 3D object counter[10]. DA neurons were manually counted through z-stacks using Cell Counter plugin using the anti-TH staining and each hemisphere represents an n number[3].

Western blotting

Drosophila heads. Quantitative Western blotting from adult fly heads were performed as previously published protocol[1]. In short, adult fly heads were lysed in RIPA buffer (Sigma) containing cOmplete proteinase inhibitor (Roche) and phosSTOP phosphatase inhibitor (Roche). The total protein concentration was measured using the BCA kit (Thermo Fisher Scientific). 10 µg protein/lane was submitted to electrophoresis in 10% SDS-polyacrylamide gel and transferred to nitrocellulose membrane. Each gel contained a control lane of pooled brain homogenates used as an internal standard in all gels. After blocking with Odyssey blocking buffer (Li-COR Biosciences), the following primary antibodies used were: anti-Synapsin (1:500 – DSHB 3C11), anti-Syntaxin (1:1000 – DSHB 8C3), anti-GFP (1:1000 - Thermo Fischer A6455), anti-beta actin (1:1000 - Abcam Ab8227), anti-beta tubulin (1:1000 – DSHB E3). Secondary antibodies were IRDye 800 conjugated goat anti-rabbit (1:10000, Rockland Immunochemicals) and Alexa Fluor 680 goat anti-mouse (1:10000, Invitrogen). Membrane images were acquired Odyssey CLx Imaging System and quantified with ImageJ.

Analysis of neuronal function

The Steady State Visual Evoked Potential (SSVEP) assay measured the output of the photoreceptors and second-order lamina neurons. On the day of eclosion, flies were placed in the dark at 29°C, 3 day-old-flies were prepared for SSVEP measurements as described[11,12]. The same protocol was used, except that stimuli were generated, and responses recorded by an Arduino Due system instead of a PC. Data was analysed in Matlab and R. Full code at https://github.com/wadelab/flyCode.

Behavioural Analyses

Drosophila ARousal Tracking (DART). DART was used to perform single fly tracking of age-matched mated females[13,14]. During each experiment, a total of 80 flies including controls and experimental genotypes were recorded. Briefly, flies were quickly anesthetised on ice and individually placed into glass tubes. The flies were allowed to recover for 30 min at 25°C prior to the beginning of the experiment. The recording was continuously performed at 5 frames per second for 2 hours using a USB-webcam (Logitech). The x/y position of every fly was tracked and analysed using DART software in order to evaluate the relative speed and activity during the recording.

Startle-induced negative geotaxis (SING). SING was used to assess the locomotor ability of flies following a startle stimulus to which flies display a negative geotaxis response (modified from Ruan et al[15]. A group of ten mated age-matched female flies, per genotype, were selected by a mouth aspirator and transferred into the experimental tubes containing 1 cm of fresh ageing food at room temperature. After the tubes of all genotypes tested being placed in custom-made apparatus (see Fig. 7A), flies were allowed to acclimatise for 20 min prior the beginning of the assay. Control and experimental groups were always assayed together by tapping all the flies to the bottom of the tubes and allowing them to climb as a negative geotaxis response. After 10 seconds, the number of flies that successfully climbed above the 7 cm line was recorded. This assay was repeated and recorded, at 30 frames per second; 5 trials were performed for each cohort and the flies were allowed to rest during 1 min between trials. The averaged data were represented as percentage. A minimum of 9 cohorts, each consisting of 10 flies (= 90 flies in total) were tested per genotype.

Proboscis extension response (PER) – Akinesia assay. The PER assay was recorded from 5-8-day-old flies. Flies were restrained as previously described[16] and starved at 25 °C for 3 hours before being offered a droplet of 100 mM sucrose three times. The proboscis extension responses were observed with a Grasshopper 3 (Point Grey) camera mounted on a Zeiss Stemi microscope at 200 frames/second. Each response was scored Yes/No and the median response for each sample used.

Human post-mortem tissue analysis

Brain tissue samples. Detailed description of brain samples, diagnose criteria and neuropathological assessments has been previously published[17]. Brain tissue samples were provided from Brains for dementia research network. Consent for autopsy, neuropathological assessment and research were obtained and all studies were carried out under the ethical approval of the regional Ethical Review Board of Stockholm (2012/910-31/4). 30 cases in total/brain regions were used for the western blot experiments. Controls were defined as subjects with no clinical history and no neuropathological evidence of a neurodegenerative condition.

Quantitative Western Blotting. For western blot analysis, 500 mg of frozen tissue was homogenized in ice-cold buffer containing 50 mM Tris-HCL, 5 mM EGTA, 10 mM EDTA, protease inhibitor cocktail tablets (Roche, 1 tablet per 50 mL of buffer), and 2 mg/mL pepstatin A dissolved in ethanol:dimethyl sulfoxide 2:1 (Sigma). Protein concentration of each sample was measured by using BCA Protein Assay Kit (Thermo Fisher Scientific). To minimize inter-blot variability, 20 μg total protein/samples were loaded in each lane of each gel on 7.5-10% SDS-polyacrylamide gel for protein separation and then transferred to nitrocellulose membrane (Immobilon-P, Millipore). After blocking, membranes were

incubated with primary antibodies followed by HRP conjugated secondary antibody. Each gel contained a control lane of pooled brain homogenates used as an internal standard. Bands were visualized using Chemiluminescent substrate (Millipore) in a LAS-3000 luminescent image reader (Fujifilm) or by using secondary antibodies compatible with the Odyssey imaging system (LI-COR Biosciences). Primary antibodies were used at the following concentrations: rabbit polyclonal anti-LIPRIN-α3 (1:1000, Synaptic Systems 169 102); Rabbit anti-LIPRIN-α4 (1:1000, Abcam - ab136305); Rabbit anti-GAPDH (1:5000, Abcam, ab22555). Secondary antibodies used were donkey anti rabbit (1:10000, Invitrogen NA9340V) or donkey anti rabbit (1:5000, LICOR, 926-32213). Western blot data were evaluated and quantified using Multi Gauge Image Analyzer (version 3.0) or Image studio Lite, respectively.

Statistical analysis

GraphPad Prism 8 was used to perform the statistical analyses. Comparison of means from 2 experimental conditions was performed using unpaired parametric two-tailed Student's t-test. Comparison of means from multiple experimental conditions was performed using ANOVA, followed by Dunnett's multiple comparison post-hoc test, when comparing the experimental groups to control only. Alternatively, when comparing all groups among each other, Tukey's multiple comparison post-hoc test was used. Samples not normally distributed were assessed by Kruskal-Wallis test followed by Dunn's multiple comparison post-hoc test. The significance was defined as $p<0.05$, error bars are shown as SEM.

References

1. Solomon DA, Stepto A, Au WH, et al. A feedback loop between dipeptide-repeat protein, TDP-43 and karyopherin-α mediates C9orf72-related neurodegeneration. *Brain*. 2018;141(10):2908-2924.

2. Diaper DC, Adachi Y, Lazarou L, et al. Drosophila TDP-43 dysfunction in glia and muscle cells cause cytological and behavioural phenotypes that characterize ALS and FTLD. *Hum Mol Genet*. 2013;22(19):3883-3893.

3. White KE, Humphrey DM, Hirth F. The dopaminergic system in the aging brain of Drosophila. *Front Neurosci*. 2010;4(December):205.

4. Friggi-Grelin F, Coulom H, Meller M, Gomez D, Hirsh J, Birman S. Targeted gene expression in Drosophila dopaminergic cells using regulatory sequences from tyrosine hydroxylase. *J Neurobiol*. 2003;54(4):618-627.

5. Poças GM, Branco-Santos J, Herrera F, Outeiro TF, Domingos PM. Alpha-Synuclein modifies mutant huntingtin aggregation and neurotoxicity in Drosophila. *Hum Mol Genet*. 2015;24(7):1898-1907.

6. Brent JR, Werner KM, McCabe BD. Drosophila Larval NMJ Dissection. *J Vis Exp*. 2009;(24):4-5.

7. Ohyama T, Verstreken P, Ly C V., et al. Huntingtin-interacting protein 14, a palmitoyl transferase required for exocytosis and targeting of CSP to synaptic vesicles. *J Cell Biol*. 2007;179(7):1481-1496.

8. West RJH, Lu Y, Marie B, Gao FB, Sweeney ST. Rab8, POSH, and TAK1 regulate synaptic growth in a Drosophila model of frontotemporal dementia. *J Cell Biol*. 2015;208(7):931-947.

9. Rao SS, Stewart BA, Rivlin PK, et al. Two distinct effects on neurotransmission in a temperature-sensitive SNAP-25 mutant. *EMBO J*. 2001;20(23):6761-6771.

10. Goel P, Li X, Dickman D. Disparate Postsynaptic Induction Mechanisms Ultimately Converge to Drive the Retrograde Enhancement of Presynaptic Efficacy. *Cell Rep*. 2017;21(9):2339-2347.

11. Afsari F, Christensen K V., Smith GP, et al. Abnormal visual gain control in a Parkinson's disease model. *Hum Mol Genet*. 2014;23(17):4465-4478.

12. Petridi S, Middleton CA, Ugbode C, Fellgett A, Covill L, Elliott CJH. In Vivo Visual Screen for Dopaminergic Rab ↔ LRRK2-G2019S Interactions in Drosophila Discriminates Rab10 from Rab3. *Genes|Genomes|Genetics*. 2020;10(6):1903-1914.

13. Faville R, Kottler B, Goodhill GJ, Shaw PJ, van Swinderen B. How deeply does your mutant sleep? Probing arousal to better understand sleep defects in Drosophila. *Sci Rep*. 2015;5:8454.

14. Shaw RE, Kottler B, Ludlow ZN, et al. In vivo expansion of functionally integrated GABAergic interneurons by targeted increase in neural progenitors. *EMBO J*. 2018;33(16):e98163.

15. Ruan K, Zhu Y, Li C, Brazill JM, Zhai RG. Alternative splicing of Drosophila Nmnat functions as a switch to enhance neuroprotection under stress. *Nat Commun*. 2015;6(1):10057.

16. Cording AC, Shiaelis N, Petridi S, Middleton CA, Wilson LG, Elliott CJH. Targeted kinase inhibition relieves slowness and tremor in a Drosophila model of LRRK2 Parkinson's disease. *npj Park Dis*. 2017;3(1):34.

17. Bereczki E, Branca RM, Francis PT, et al. Synaptic markers of cognitive decline in neurodegenerative diseases: a proteomic approach. *Brain*. 2018;141(2):582-595.
